# Loss‐of‐function coding variants in the Ras of complex proteins/GTPase domain of leucine rich repeat kinase 2

**DOI:** 10.1002/pro.70190

**Published:** 2025-06-22

**Authors:** Sarah Butterfield, Susanne Herbst, Patrick Alfryn Lewis

**Affiliations:** ^1^ Department of Comparative Biomedical Sciences Royal Veterinary College Camden Town UK; ^2^ Department of Neurodegenerative disease UCL Queen Square Institute of Neurology London UK

**Keywords:** GTPase, leucine rich repeat kinase 2, LRRK2, lysosomes, Parkinson's

## Abstract

The *LRRK2* gene is a key contributor to the genetic risk of Parkinson's disease, and a priority drug target for the disorder. Leucine Rich Repeat Kinase 2, the protein product of *LRRK2*, is a multidomain enzyme implicated in a range of cellular processes—including endolysosomal trafficking and damage response. Based on the report that truncation and structural variants resulting in loss of LRRK2 protein are observed in human populations, genomic sequence repositories were queried for coding variants affecting key catalytic residues in LRRK2—resulting in the identification of three variants (K1347E, K1347R, and T1348P) predicted to ablate the capacity of LRRK2 to bind GTP. Biochemical and cellular characterization of these variants confirmed loss of GTP binding, as well as reduced or loss of kinase activity. These data demonstrate the presence of rare coding enzymatic loss‐of‐function variants in humans, with implications for our understanding of LRRK2 as a driver of disease and as a drug target.

## 1 INTRODUCTION

Parkinson's disease (PD) is a common neurodegenerative disease characterized by autonomic dysfunction, progressive movement disorder, and cognitive decline. This is coupled with neuronal loss and the accumulation of alpha synuclein in intracellular inclusions called Lewy bodies (Morris et al., 2024). Whilst idiopathic cases of multifactorial etiology represent the majority of cases, around 5%–10% of PD is associated with a familial pattern of inheritance (Westenberger et al., 2024). Of these familial cases, autosomal dominant coding mutations in the *LRRK2* gene on chromosome 12 are the most common cause, with more common coding and non‐coding variants at the *LRRK2* locus also linked to increased lifetime risk of developing idiopathic Parkinson's (Kluss et al., 2019).

Leucine Rich Repeat Kinase 2 (LRRK2), the product of the *LRRK2* locus, is a multi‐domain scaffold enzyme that possesses both guanosine triphosphatase (GTPase) and kinase activities (Alessi & Pfeffer, 2024). The catalytic core of LRRK2 is composed of the ROCO/GTPase supradomain (a Ras of complex proteins [ROC] domain and a C‐terminal of ROC [COR] domain), followed by a serine–threonine kinase domain. Notably, PD associated coding mutations in LRRK2 cluster within the enzymatic domains of the protein and include the N1437H, R1441C (both in the ROC/GTPase domain), Y1699C (in the COR domain), and G2019S and I2020T mutations (both in the kinase domain). These variants act by distorting the enzymatic activities of LRRK2, increasing kinase activity and decreasing GTPase activity (Zhu et al., 2023). LRRK2 has been reported to directly phosphorylate a number of substrates, in particular a subset of Rab GTPases (Steger et al., 2016, 2017). Mutations result in increased phosphorylation and disruption of intracellular trafficking—although the mechanisms connecting these events to neurodegeneration remain obscure (Taymans et al., 2023). The prevalence of *LRRK2* mutations and the enzymatic activities of the protein have established LRRK2 as a leading target for drug discovery, with ongoing clinical trials for kinase inhibitors and antisense oligonucleotides (Lewis, 2022).

One of the many intriguing aspects of the molecular genetics of *LRRK2* is the identification of loss‐of‐function (LOF) variants at the *LRRK2* locus (Blauwendraat et al., 2018). Initially identified through a PD case/control study, large‐scale analysis of the *LRRK2* locus from the Gnomad, 23andMe, and UK Biobank cohorts revealed 1455 individuals with LRRK2 heterozygous LOF variants, including frameshifts, premature stop codons, and alterations in splicing, with a population frequency of 0.48% (Whiffin et al., 2020). No evidence of association of heterozygous *LRRK2* LOF with disease has yet emerged, despite a significant reduction in LRRK2 protein levels.

Given the prominence of the enzymatic activities of LRRK2 in its function and disease association, the increasing comprehension of the structure/function of this protein and proliferation of exome sequence data across human populations provides an opportunity to test whether there are specific coding variants that result in loss of enzymatic function of LRRK2—the subject of this current study.

## 2 RESULTS AND DISCUSSION

### 2.1 Naturally occurring LRRK2 GTP‐binding site mutants lose the ability to bind GTP

**FIGURE 1:**
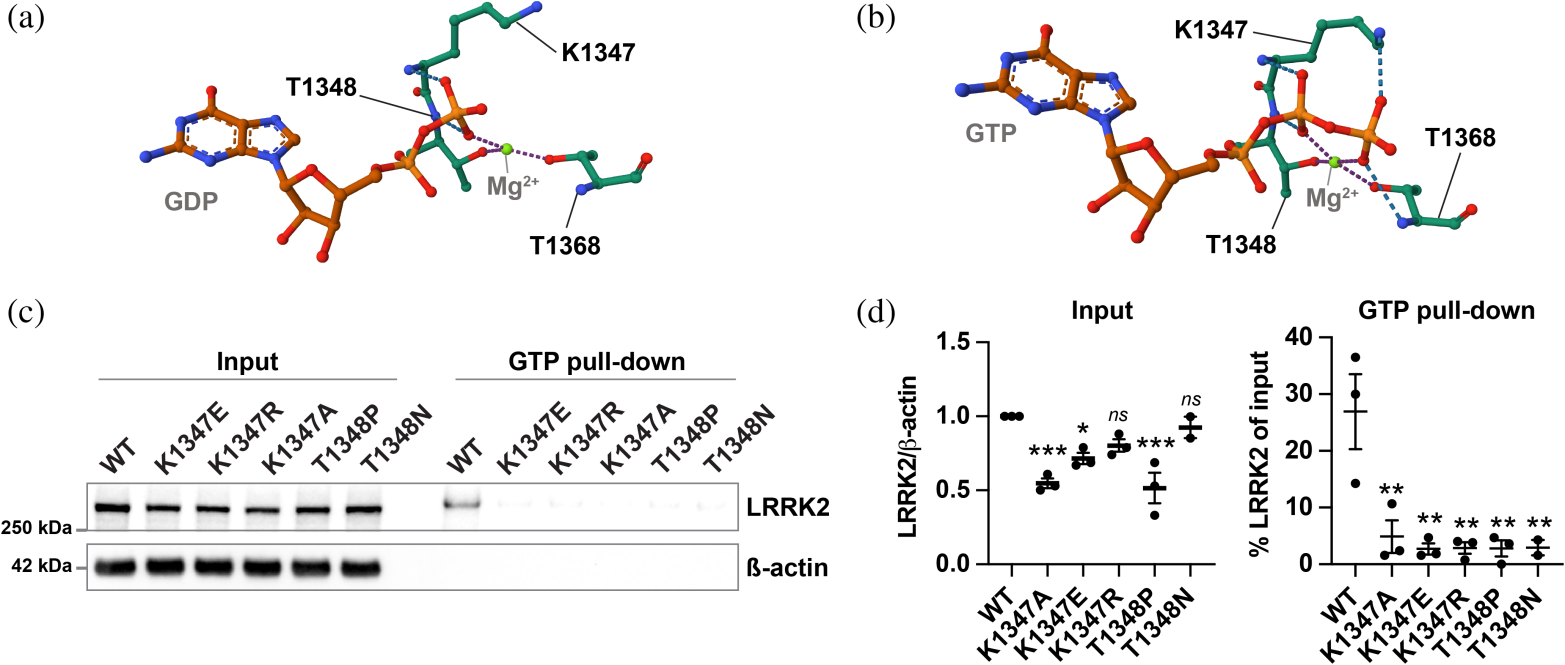
Naturally occurring LRRK2 GTP‐binding variants lose the ability to bind GTP. (a) Contribution of K1347 (Lys1347) and T1348 (Thr1348) to GDP binding in a LRRK2 AlphaFold3 model, containing Mg^2+^. (b) Contribution of K1347 (Lys1347) and T1348 (Thr1348) to GTP binding in a LRRK2 AlphaFold3 model, containing Mg^2+^. (c) LRRK2 GTP‐agarose pull‐down of artificial and naturally occurring K1347 and T1348 LRRK2 variants. HEK293 cells were transfected with the indicated LRRK2 constructs, and LRRK2 was pulled down from the cell lysates using GTP‐agarose. WBs of the cell lysate (1% of pull‐down) and the pull‐down were analyzed by anti‐LRRK2 staining. (d) Quantification of total LRRK2 levels from the input and the % of pulled down LRRK2. *N* = 2–3. One‐way ANOVA, followed by Dunnett's multiple comparisons test against WT. *ns*—not significant, *p > 0.05, **p > 0.01, ***p > 0.001.

The critical importance of residues K1347 and T1348 in the active site of the ROC domain of LRRK2 for GTP‐binding and hydrolysis has previously been reported, with artificial mutations at these codons (K1347A and T1348N) ablating the capacity of LRRK2 to bind GTP (Ito et al., 2007; Lewis et al., 2007). As the current LRRK2 full‐length structures lack the resolution to identify densities for Mg^2+^, which plays a major part in the GTPase cycle (Myasnikov et al., 2021; Vetter & Wittinghofer, 2001), we modeled LRRK2 in a GDP or GTP‐bound state in the presence of Mg^2+^ using AlphaFold3. In accordance with the loss of GTP‐binding of the K1347A and T1348N mutants, these models predict that residue K1347 co‐ordinates the binding of the *β*‐phosphate of GDP and GTP (Figure 1a, b) and directly interacts with the *γ*‐phosphate of GTP (Figure 1b). They further predict that T1348 coordinates the Mg^2+^ that contributes to the positioning of the *β*‐phosphate of GDP and the *β*‐phosphate and *γ*‐phosphate of GTP (Figure 1a, b). To identify naturally occurring putative LRRK2 GTP‐binding null mutants, the gnomAd and MCPS Variant Browser were accessed to search for variation at these residues, resulting in the identification of heterozygous carriers of K1347E, K1347R, and T1348P variants (Table S1) (Karczewski et al., 2020; Sun et al., 2024).

To test the impact of these naturally occurring ROC domain variants on GTP‐binding, these mutations were inserted into the full‐length LRRK2 open reading frame, alongside the previously characterized GTP‐binding deficient artificial mutants K1347A and T1348N. These mutants displayed robust expression in HEK293 cells, although some of the GTP‐binding variants displayed decreased expression levels when compared to WT (Figure 1c, d). GTP‐binding was assessed by GTP‐agarose bead pull‐down. When compared to input levels, only WT LRRK2 was pulled down using GTP‐agarose beads (Figure 1c, d), indicating that the naturally occurring LRRK2 GTP‐binding site mutants have a greatly reduced affinity for GTP.

### 2.2 Naturally occurring LRRK2 GTP‐binding site mutations reduce kinase function

As the LRRK2 GTPase and kinase functions are highly interlinked, we next investigated the impact of the LRRK2 GTP‐binding site mutants on LRRK2 kinase activity. The GTPase mutants were expressed in HEK293 cells, and LRRK2 kinase activity was assessed in the presence and absence of the lysotoxic agent LLOMe (which stimulates LRRK2 activity) by measuring the phosphorylation of the LRRK2 substrates Rab10 and Rab12, and LRRK2 autophosphorylation at S1292.

**FIGURE 2:**
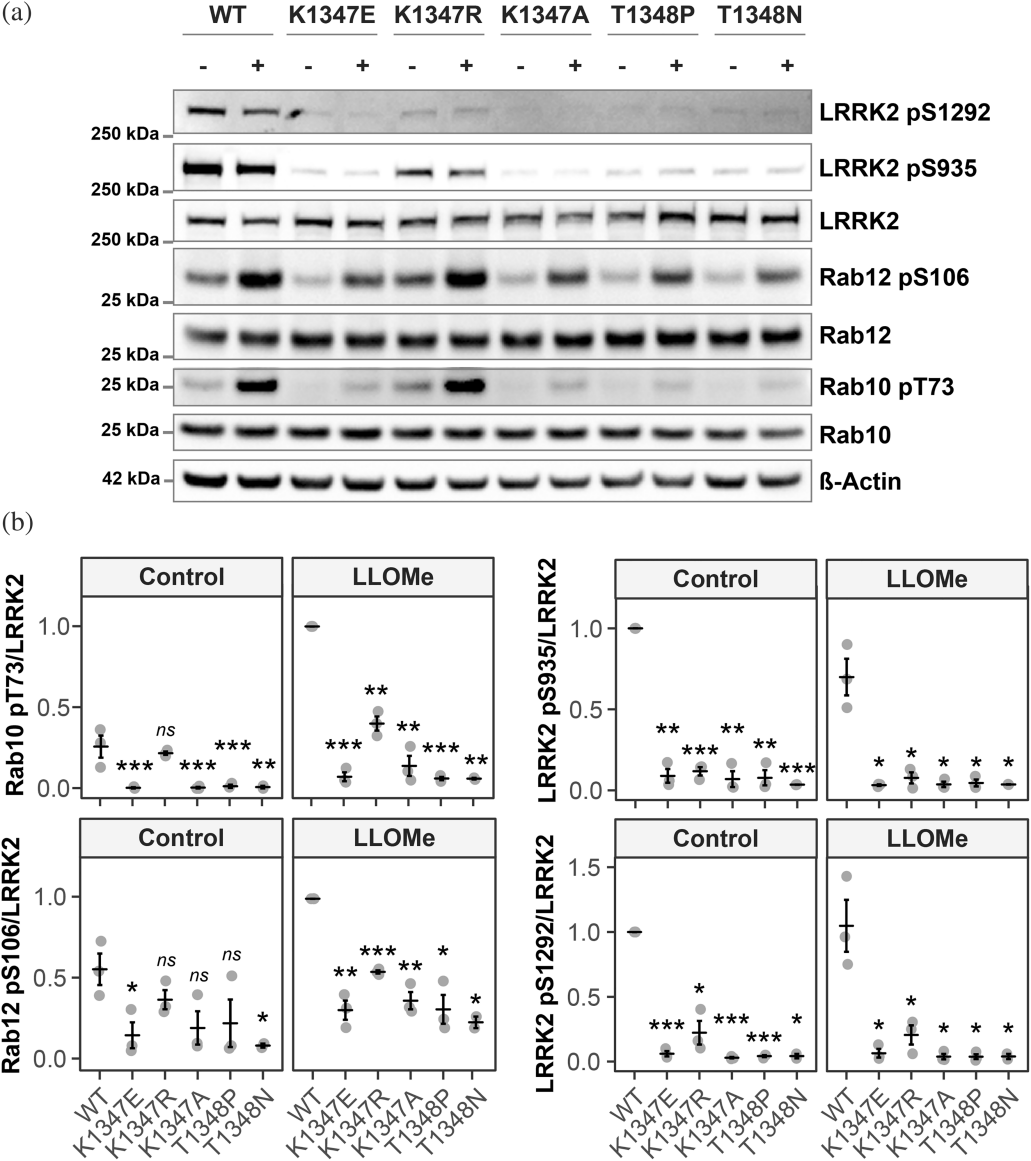
LRRK2 GTP‐binding variants impair LRRK2 kinase activity. HEK293 cells were transfected with the indicated EGFP‐LRRK2 GTP‐binding variants and treated with 1 mM LLOMe for 1 h where indicated. (a) LRRK2, Rab10, and Rab12 phosphorylation was assessed by Western blot. (b) Quantification of Rab10 T73 and Rab12 S106 phosphorylation (normalized to WT LLOMe) and LRRK2 S935 and S1292 phosphorylation (normalized to WT control) from (a). *N* = 3. Statistical analysis was conducted by two‐way ANOVA followed by a *t*‐test corrected for multiple comparisons against WT. *ns*—not significant, *p > 0.05, **p > 0.01, ***p > 0.001.

**FIGURE 3:**
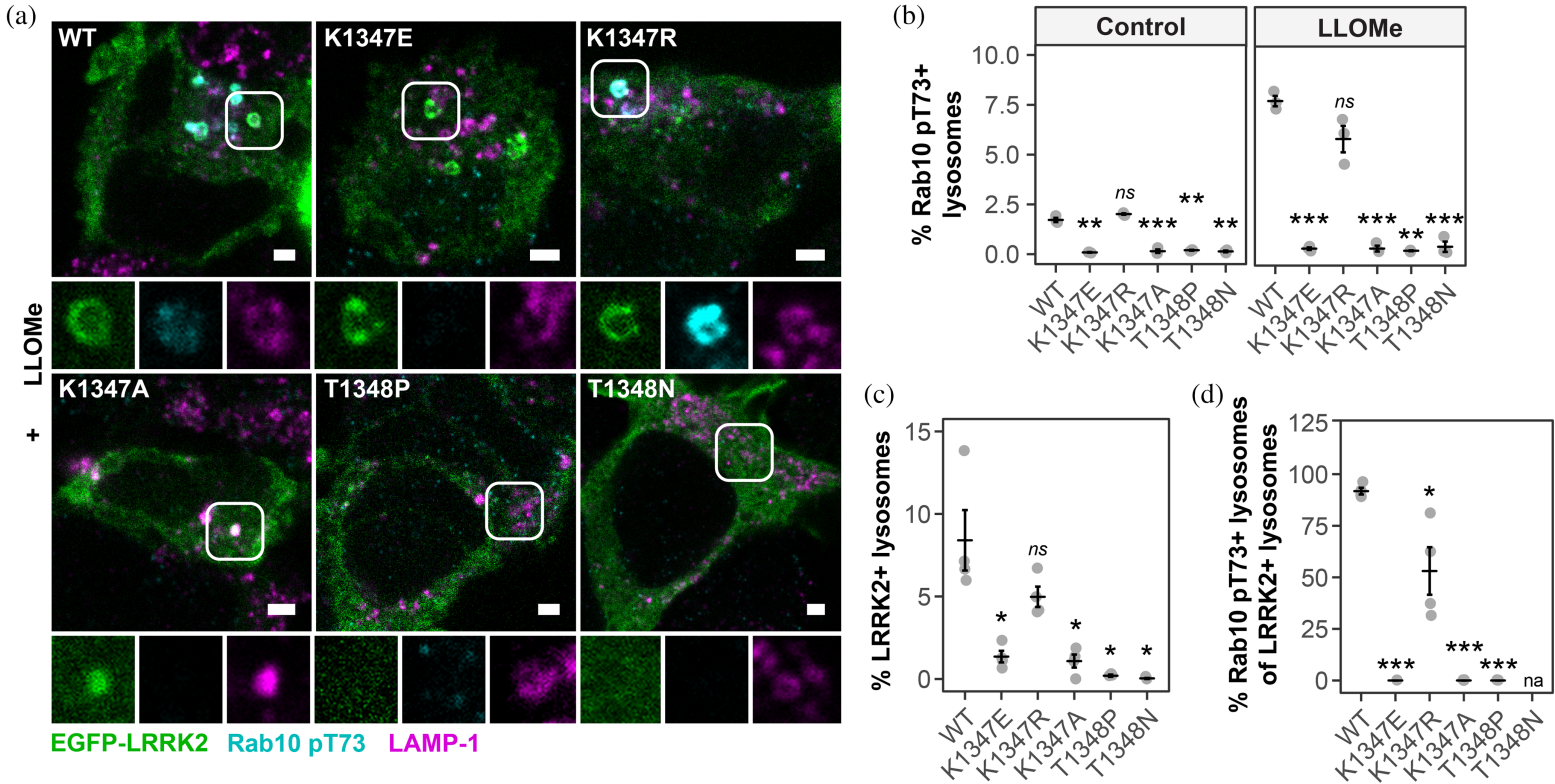
LRRK2 GTP‐binding variants impair LRRK2 lysosomal recruitment. HEK293 cells were transfected with the indicated EGFP‐LRRK2 GTP‐binding variants and treated with 1 mM LLOMe for 1 h. (a) Co‐localization of EGFP‐LRRK2, Rab10 pT73, and LAMP‐1 was assessed by high content imaging. Scale bar = 5 μm. (b) Quantification of the % of Rab10 pT73+ lysosomes from (a) *n* = 3. (c) Quantification of the % of LRRK2+ lysosomes from (A) *n* = 4. (d) % Rab10 pT73+ lysosomes of LRRK2+ lysosomes from (a), *n* = 4. Statistical analysis was conducted by two‐way ANOVA followed by a t‐test corrected for multiple comparisons against WT. ns—not significant, *p > 0.05, **p > 0.01, ***p > 0.001. na*—*not applicable as no LRRK2+ lysosomes were detected.

At both steady‐state and following LLOMe stimulation, the K1347E and T1348P mutants displayed a marked reduction of kinase activity as evidenced by a loss in Rab10 and Rab12 phosphorylation, and a concomitant decrease in pS1292 phosphorylation of LRRK2. In contrast, and surprisingly, the K1347R mutant retained partial kinase activity (Figure 2a, b). To further test whether the GTP‐binding site mutant LRRK2 is recruited to lysosomes and is capable of activating Rab proteins following damage, cells expressing these variants were treated with LLOMe, and the response of LRRK2 assessed using fluorescence microscopy. Analysis of Rab10 pT73 activation and localization to lysosomes mirrored the results obtained by Western blot, with only the K1347R variant retaining Rab10 T73 phosphorylation (Figure 3a, b). Although greatly reduced, we were still able to detect lysosomal recruitment of LRRK2 K1347 mutants whereas we did not detect any recruitment of the LRRK2 T1348 mutants (Figure 3a, c). Of note, however, despite residual recruitment, we did not detect any Rab10 pT73 signal for the K1347E and K1347A variants whereas the K1347R variant was still able to phosphorylate Rab10 (Figure 3a, d). As mutations at T1348 are predicted to result in a complete loss of nucleotide binding, this finding may indicate that GDP‐loaded LRRK2 can still be recruited to membranes and GTP‐binding constitutes a requirement for kinase activity.

Taken together, these data suggest that naturally occurring variation at residues required for LRRK2 GTPase function leads to a de facto loss of LRRK2 GTP binding, resulting in ablation or a reduction in kinase activity. Whether it is the reduction of GTP hydrolysis or the inability to bind guanosine nucleotides per se that reduces kinase activity is not clear. This observation expands the range and nature of LOF variants observed in LRRK2 and highlights the need to consider coding variants as well as frameshift or premature stop codon mutations in the context of reduced LRRK2 activity.

No clinical phenotype has been reported with the K1347E, K1347R, or T1348P variants from the genomic repositories, and to date none of these have been identified in screens for disease‐associated mutations. Notably, the loss of enzymatic activity—while retaining scaffolding function—more closely matches the cellular consequences of treatment with small molecule LRRK2 kinase inhibitors compared to allelic variants resulting in complete loss of protein. Given that the majority of PD‐associated mutations result in increased LRRK2 kinase activity, it is unlikely that the ROC domain LOF variants would predispose carriers to Parkinson's, and may even reduce the risk of disease.

An intriguing, and perhaps perplexing, aspect of these results is the observation that the K1347R variant retained the ability to phosphorylate Rab substrates (albeit at a reduced level compared to WT), despite not being able to bind GTP‐agarose. This discrepancy could be due to the GTP‐agarose bead pull‐down lacking sensitivity, or being limited to cellular lysates rather than performed in a living cellular environment, and not detecting residual GTP binding capacity for this variant. It is of note that the arginine side chain retains a positive charge, similar to the wild type lysine, although the increased size of the arginine side chain could result in steric hindrance of GTP binding.

In summary, this study reports the existence and characterizes the consequences of LOF coding variants in the ROC/GTPase domain of LRRK2. This highlights the need to consider naturally occurring functional coding variants in the context of LRRK2 activity and supports further investigation into whether these variants act to increase or decrease the risk of disease.

## AUTHOR CONTRIBUTIONS

**Sarah Butterfield:** Investigation; writing – original draft. **Susanne Herbst:** Conceptualization; investigation; validation; supervision; writing – review and editing. **Patrick Alfryn Lewis:** Conceptualization; funding acquisition; writing – review and editing; supervision.

## CONFLICT OF INTEREST STATEMENT

PAL is a paid consultant for Serna Bio.

## Supporting information


Data S1

Data S2

## Data Availability

The data that supports the findings of this study are available in the supplementary material of this article.
