# Supplementary figures and images for: Loss‐of‐function coding variants in the Ras of complex proteins/GTPase domain of leucine rich repeat kinase 2
Source: Protein Sci. 2025 Jun 22;34(7):e70190. doi: 10.1002/pro.70190 (PMC12183101; doi:10.1002/pro.70190)

Figure 1

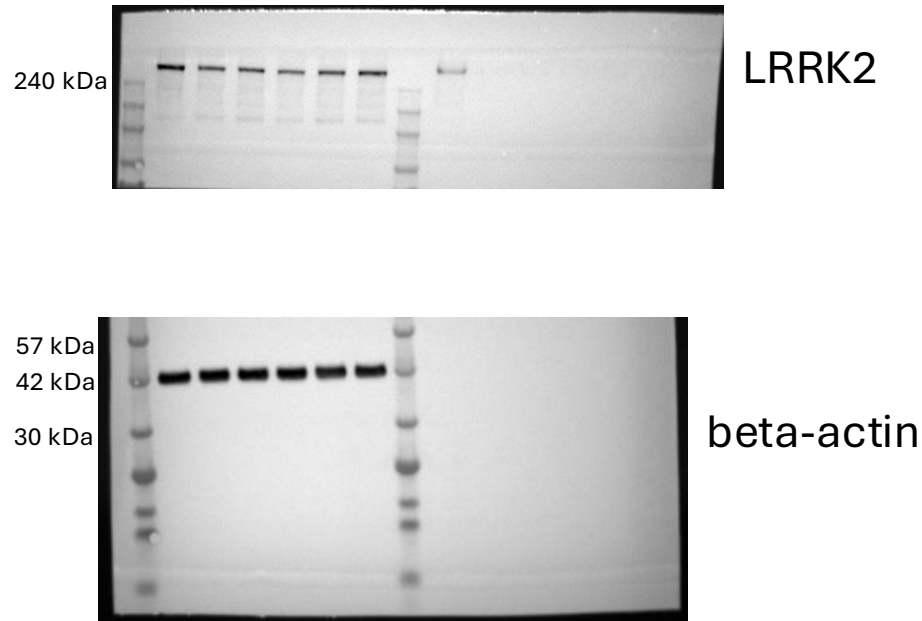

Figure 2

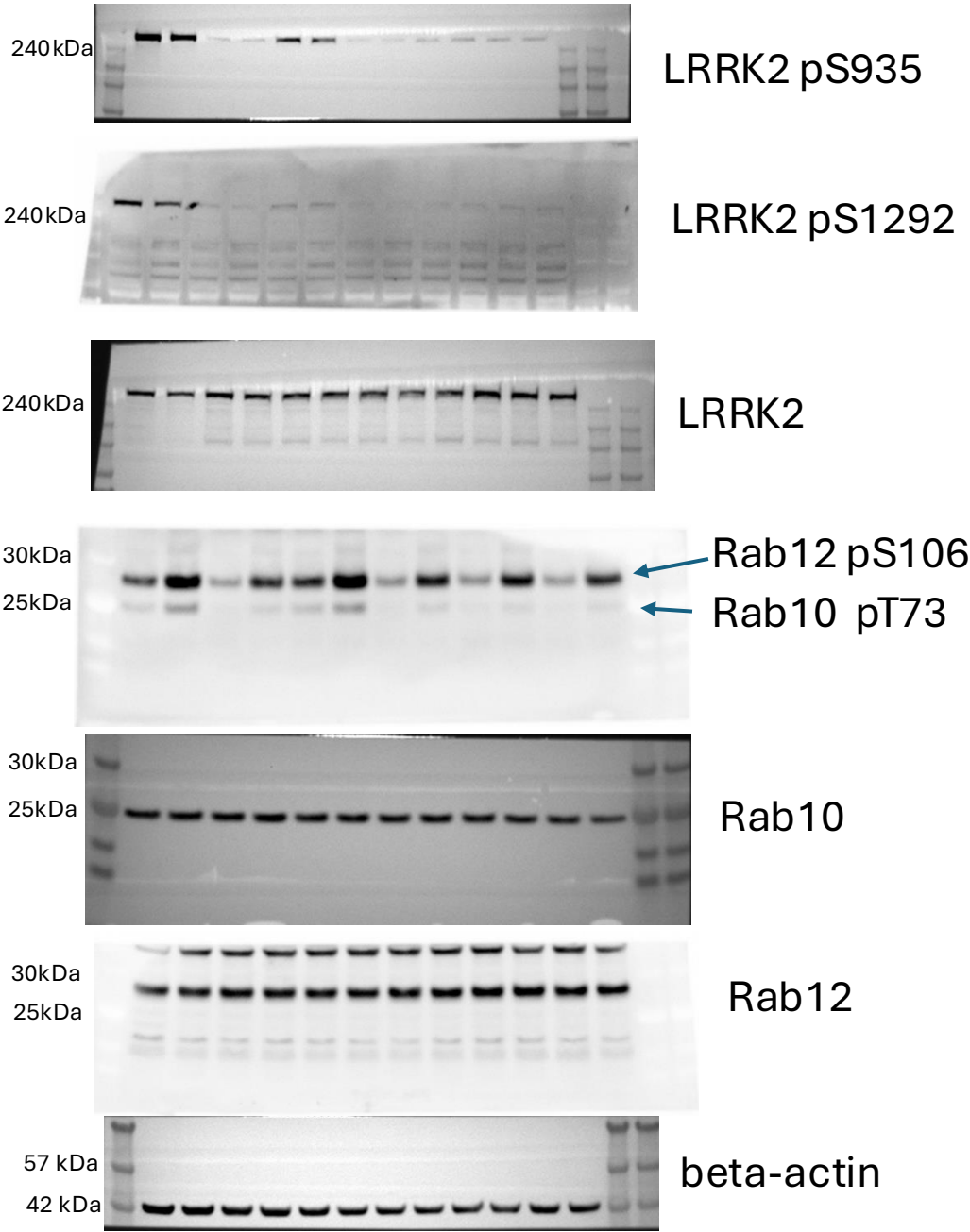

Supplement: Supplementary file 1 — Data S1 [file PRO-34-e70190-s002.pdf]
