# Supplementary material for: Loss‐of‐function coding variants in the Ras of complex proteins/GTPase domain of leucine rich repeat kinase 2
Source: Protein Sci. 2025 Jun 22;34(7):e70190. doi: 10.1002/pro.70190 (PMC12183101; doi:10.1002/pro.70190)
Supplement: Supplementary file 2 — Data S2 [file PRO-34-e70190-s001.docx]

| **Variant** | **Consequence** | **Allele Frequency** | **Source** |
| --- | --- | --- | --- |
| 12:40308546:A:G | K1374E | 7.23e-6 | MCPS Variant browser |
| 12:40308547:A:G | K1374R | 6.20e-7 | gnomAD browser |
| 12:40308549:A:C | T1348P | 1.86e-6 | gnomAD browser |
| *Table S1: LRRK2 GTP-binding variants identified in the MCPSV and gnomAD variant browsers.* | | | |
